# Digital Health and the State of Interoperable Electronic Health Records

**DOI:** 10.2196/12712

**Published:** 2019-11-01

**Authors:** Jessica Germaine Shull

**Affiliations:** 1 PhD Program, Department of Biomedicine University of Barcelona Barcelona Spain; 2 Institute for Biomedical Research Bellvitge Hospital Bellvitge Barcelona Spain

**Keywords:** EHRs, health information technology, machine learning in health

## Abstract

Digital health systems and innovative care delivery within these systems have great potential to improve national health care and positively impact the health outcomes of patients. However, currently, very few countries have systems that can implement digital interventions at scale. This is partly because of the lack of interoperable electronic health records (EHRs). It is difficult to make decisions for an individual or population when the data on that person or population are dispersed over multiple incompatible systems. This viewpoint paper has highlighted some key obstacles of current EHRs and some promising successes, with the goal of promoting EHR evolution and advocating for frameworks that develop digital health systems that serve populations—a critical goal as we move further into this data-rich century with an ever-increasing number of patients who live longer and depend on health care services where resources may already be strained. This paper aimed to analyze the evolution, obstacles, and current landscape of EHRs and identify fundamental areas of hindrance for interoperability. It also aimed to highlight countries where advances have been made and extract best practices from these examples. The obstacles to EHR interoperability are not easily solved, but improving the current situation in countries where a national policy is not in place will require a focused inquiry into solutions from various sources in the public and private sector. Effort must be made on a national scale to seek solutions for optimally interoperable EHRs beyond status quo solutions. A list of considerations for best practices is suggested.

## Introduction

Digital health systems and innovative care delivery within these systems have great potential to improve national health care and positively impact the health outcomes of patients. However, currently, very few countries have systems that can implement digital interventions at scale. This is, in part, because of the well-known lack of interoperable electronic health records (EHRs). EHRs are the connective tissue of a health system; yet, most countries have systems that cannot unite the information of all their citizens because even within 1 city, the software used in 1 hospital is incompatible with that used in another, although it may have been procured from the same company.

Once digital health records are interoperable, digital health systems may function on a national scale rather than piecemeal or in isolation. Treatment of noncommunicable diseases could benefit from digital health coaching, personalized delivery of care, and quality of care improvements [1-3]. In addition, there may be thousands of patients who share the symptoms or side effects or respond in revealing patterns, leading to new treatments and personalized medicine, and even new disease surveillance tools could be developed [4]. However, we cannot explore or create these tools without aggregating and sharing patient data under a common set of (secure) standards. Without interoperability, using these tools or implementing remote patient monitoring products is extremely complex or simply happens in small samples. This potential use of health information technology has been discussed in papers dating back to 2005 [5] and analyzed for impact once EHRs were well established [6]; however, because of legacy systems and the design of those systems, the choices of EHRs are limited and incapable of algorithmic analysis across their entire client base. Epic (Epic Systems Corporation), for example, is an EHR software first designed in 1979 [7], and although the code has evolved since then, the company commands a 10-fold price difference compared with more agile solutions with no evident improvement in quality [8]. Epic is simply the most prevalent EHR available in the United States; the company now archives at least part of the health records of approximately half the US population [8].

Partners HealthCare is a sprawling 10-hospital system in Boston, which, in 2015 to 2016, spent US $1.2 billion [9] implementing an upgraded EHR system from Epic. The intention was to decrease errors and unify a previously disparate network that made it difficult for physicians in 1 hospital within the system to cross-reference information from a patient who had been at another [10]. When the system launched, 1000 Epic employees were required on hand to troubleshoot, which was perhaps foreseen, but the technical issues that persist today may not have been calculated. As recently as February 2019, the system was down for several hours [11], and it does not perform integration that patients are beginning to expect, such as integrating data from a connected glucose monitor. The upgrade was simply not designed with this feature. This integration is possible with a consolidation of the Apple HealthKit and the Dexcom Share2 app, as piloted on an Epic system in 2016 [12], but this extra patching work perhaps should not be required with a billion-dollar price tag. Epic is by no means alone on the list of EHR companies that are faced with a more technically savvy client base who expect better interfaces and services, and they are trying to become more agile; in 2018, the company announced One Virtual System Worldwide, which allows providers to access patient information from other institutions [13], which is a notable step but perhaps not on par with the data integration and analysis capabilities of companies such as Google and Apple.

Digital health investment is at an all-time high, with nearly US $7 billion invested in 2018 in the United States alone [14]. However, most of the solutions being funded are not addressing the administrative foundations of the health care system such as EHRs. Investment in artificial intelligence (AI) for health care, a perhaps more tantalizing venture, is estimated to reach US $6.6 billion by 2021 [15]. Meanwhile, the current state of real workflows at hospitals is not sustainable.

Physicians in the United States may spend half their day filling out patient histories. A 2018 study in Family Medicine found that of 982 patient visits that each lasted on average for 35.8 min, 19.3 min were spent on the EHR [16]. It is imperative that health systems gain time (and quality) where they can. In Spain, there is a notable delay to health care access; the Spanish Ministry of Health reported that in 2018, wait times for surgery had improved but were still 137 days in certain regions [17]. Systems subject to a lack of efficiency cannot afford more time, and valuable analysis is lost because of poor information systems.

## Machine Learning With Electronic Health Records

AI is often presented as a solution to ease some of this burden. In a recent paper from Cold Spring Harbor Laboratory, numerous AI and machine learning (ML) opportunities in medicine were outlined and discussed. Among those of note were methods to classify patients according to the tests that doctors ordered for them: “Perhaps deep neural networks, by reevaluating data without the context of our assumptions, can reveal novel classes of treatable conditions [18].*”* To do this, however, the AI must be carefully taught with data, much or all of it coming from EHRs, that are accurate and standardized. The term *garbage in, garbage out*, attributed most often to George Fuechsel [19], encapsulates the biggest issue with AI and ML. The outputs of the system will inevitably reflect the quality and biases of the data fed into it. At a recent AI hackathon for health outside Barcelona in 2018, the Medical Information Mart for Intensive Care (MIMIC) database was used. It is a freely accessible database that has associated more than 53,423 admissions at a large hospital in Boston from the years 2001 to 2012 [20]. The MIMIC, MIMIC II, and MIMIC III datasets have been used numerous times to demonstrate health analytics, and explorations of ML predicted patient outcomes. However, it was stated at the event that the data took nearly 2 years to *clean* and this length of time is not realistic if we have to use historical and real-time data to treat patients in the present. For MIMIC, creating an interoperable database was complex; standards on how to indicate fluid intake, data from critical care information systems, and data from the Social Security Administration all had to be developed, and this was within a single hospital system.

There is no formula for the exact sample size needed in ML, although the more complex the problem, the more data needed [21]; more complex questions such as disease treatment decisions carry a high level of risk; therefore, 100,000 could be considered a reasonable starting point. If the target is to analyze a specific rare disease, such as idiopathic pulmonary fibrosis, EHRs from multiple sources would have to be accumulated to reach that number, as the disease affects 18 of every 100,000 adults [22]. Extrapolating from these statistics, if we had complete EHRs from, for example, the entire state of Texas with a population of 28.7 million, we may get information on 5000 patients—which is not nearly enough. To effectively analyze health records from patients with rare diseases and to identify indicators within those populations, the ML would need to be able to *read* EHRs from across the United States (population 328,929,623 as on May 23, 2019 [23]) and ideally aggregate data from other countries as well.

We are at the beginning of a data-rich and connected century. To deliver optimal care to the millions of patients who are living longer with more complex and chronic diseases, we need to harness the fundamental technology of interoperable EHRs.

## Obstacles

### Cost

Health care expenditure for the entire country of Spain was more than €65 billion in 2015, according to a European Observatory on Health Systems and Policy report [24]. A US $1.2 billion expenditure (as occurred at Partners HealthCare) to integrate 1 hospital system is not feasible in countries with fewer resources, as is the case in many single-payer systems. Solutions that do not financially cripple a health care system need to be identified. There are now companies that are harnessing the immense value of health data and are willing to implement integration and analysis systems at no cost in return for access to data [25]. This is a new frontier, akin to the new marketplace of genomic data, which likely has its own set of benefits and repercussions, and it must be analyzed as to what the implications will be.

The cost, however, should be calculated by subtracting the added value of the benefits. With properly integrated EHRs, administrative costs can be lowered, adherence rates to care protocols have shown to improve [26,27], and many in-patient visits could be achieved with remote monitoring or telehealth services. In addition, ideally, the software could scan for coding errors, which are also costly in the United States (see the section Coding and Semantics).

### Coding and Semantics

The main technical issue with arriving at interoperability is the huge variation in semantics and coding standards. Hospitals code their patients differently; in our hospital, we use a case number 7 digits long, but a neighboring hospital uses their own system with 6 digits. There is a unique identifier for each patient in the regional system, but many hospitals do not enter that data at all, and that regional identifier is not used at the national level.

The Logical Observation Identifiers Names and Codes (LOINC) is an international standard used by more than 78,000 agencies and health care institutions to code for health measurements and observations [28]. However, in everyday practice, these standards are not used in EHRs. Blood pressure, for instance, in the United States, will be documented as 120/80 mm Hg. LOINC states, “They should be reported as 2 separate variables, systolic (LOINC 8480-6) and diastolic (LOINC 8462-4) [29].” That same reading will be noted as 12/8 mm Hg in some European countries. In addition, blood sugar is annotated differently across borders. In the United Kingdom, blood sugar is annotated in millimoles per liter. A normal reading would be *under 7.8* [30]. In the United States, blood sugar is usually written using milligrams per deciliter; therefore, a normal reading is 70 to 130 mg/dL. These are simplistic examples to illustrate a fundamental concept.

There is also the conundrum of free text. Health care professionals annotate data and events in different ways. In our hospital, arterial hypertension may be listed in more than 4 ways: ht, HTA, hypertension, or hipertensió. In addition, text is used for describing symptom and disease evolution as well as test results. All these would have to be standardized or interpreted to make logical comparisons between charts. There has been an increased use of natural language processing to read free text in an EHR for disease phenotyping [31] and even detecting associations that led to adverse events [32], and it is likely this technology will be applied on a broader scale as it improves and becomes automated.

Human error is also a consideration. Within 1 country, there may be discrepancy among codes for diagnosis. When International Classification of Diseases (ICD), Tenth Revision, Clinical Modification (ICD-10-CM) replaced ICD-9 in the United States in 2015, the coding options increased 10-fold, from 14,400 to 144,000 [33]. The ICD-10-CM codes were linked to reimbursement for health care services, which made it all the more critical that codes be correct because mistakes could be taken as fraud. The Centers for Medicare and Medicaid Services released data indicating preventable billing errors had cost US $31.6 billion in 2018 [34]. However, it has been found that 1 ICD-9 code could be interpreted as 100 different ICD-10-CM codes, and not all of these codes seem logical: Y92.241, hurt at the library; W56.22, struck by Orca, initial encounter [33]. The United States is the only country that uses ICD-10-CM, creating yet another layer of incompatibility. If we had global compatibility of ICD coding, the statistics for global health would be far more accurate, which could, in theory, shift treatment protocols by allocating resources more precisely or seeing new trends in both communicable and noncommunicable diseases.

### Privacy Issues

Privacy and security for health care data are of utmost importance. Effort must be made to educate health care administrations on how EHRs work, why they can be considered as safe as banking data, and what cybersecurity checks are in place and emphasize the importance of a continually updated security plan. Often, it is not a real security risk that needs to be addressed but the perception of risk [35]. Blockchain or other technologies should be analyzed for use, and more importantly, personnel who are equipped to detect and patch issues as well as develop solutions should be on staff.

### Analysis of Progress

Over the years, there have been substantial efforts in the advocacy for EHR systems to integrate their internal sources of data as well as myriad external sources of patient information. Exemplary work from Mandl and Kohane in 2009 petitioned for EHRs and personally controlled health records to be *built on open standards, accommodating both open-source and closed-source software*, including data generated by a patient’s iPhone [36]. The authors advocated as well for federal support to clear the financial and taxonomic barriers to achieve this asking, “Can we produce a medication list for every American that can be obtained through standards-based, interoperable, substitutable applications?” The answer then was no, but open standards efforts are currently being developed and used internationally.

For instance, there is now RxNorm from the US National Library of Medicine, which can *mediate messages between systems not using the same software and vocabulary*, linking names of clinical drugs and drug interaction software [37].

Fast Health Interoperability Resources (FHIR) from HL7 is now widely recognized as the standard for EHR integration; it is used by Google for its Cloud Healthcare API stating that “FHIR specifies a robust, extensible data model for interacting with clinical resources” [38]. Analysis of data from Centers for Medicare & Medicaid Services and the Office of the National Coordinator for Health Information Technology in 2018 revealed that 32% of health information technology developers in the United States are using 2015 FHIR-certified standards, and the biggest EHR companies (including Epic and Cerner) are to some extent using FHIR standards [39]. Microsoft announced their Azure API for FHIR in February 2019 [40], and FHIR standards are also being used for the integration of wearables data and personalized devices [41]. SMART for FHIR is a project, which started in 2010 (FHIR was defined during the project) at Harvard Medical School and Boston Children’s Hospital, aimed for medical applications to run without modifications across disparate health information systems. Mandel et al. demonstrated that within 2 months, a couple of software engineers could implement SMART on FHIR for 4 different EHR vendors [42].

In January 2018, Apple announced their version of a personalized EHR called HealthKit [43], which patients can access on their iPhone; it would appear Apple understands the value of providing a service that is user-friendly and that patients can monitor themselves and integrate data from fitness devices that connect to Apple.

## Examples of Innovation in National Electronic Health Record Systems

Most countries in the world now use digital health records to some extent. The author of this paper (JS) collaborated with a team from the World Health Organization that was implementing a digital health information system in Sierra Leone in 2007, chosen precisely because it was a nearly entirely paper-based system and therefore a blank canvas. Since then much more infrastructure has been installed, and Sierra Leone has implemented district health information software from HISP in large hospital centers; in 2016, during the Ebola outbreak, a specialized EHR based on OpenMRS was developed for the Ebola treatment centers [44].

Rwanda began implementing OpenClinic electronic medical record in 2007, and it is now used throughout the country, in 20 hospitals and clinics [45]. In 2016, the Rwandan Ministry of Health partnered with Babylon Health, a company that now offers electronic prescriptions and telephone consultations to the now more than 2 million subscribers [46]. In addition, users can access their clinical records anytime via their phone, including images and audio and video of the consultations.

Estonia is another country where significant advances in health information technology innovation have been deployed at scale. The government launched an effort in 2016 to implement blockchain validation into the national EHR [47], the first country in the world to do so. The technology ensures data integrity and substantially reduces the risk of malicious intent or hacking because of blockchain’s immutable data logs. This addresses the aforementioned issue of security, often cited by health care administrations when the question of electronic health data sharing is discussed.

In 2016, the Thai Health Information Standards Development Center published a plan for adopting national standards for patient health care summary, laboratory terminology (LOINC), syntax (HL7), and security (MICT) [48]. Thailand has already been notably forward-thinking by creating a unique national identifier system and achieving universal health care coverage in 2002 [49]; hence, the country is familiar with the effort it takes to align all the stakeholders involved in this type of initiative.

Israel has an integrated health monitoring system covering 4.2 million patients [50]. Since the implementation, studies have shown that patients are more adherent to medications [51].

In January 2019, Abu Dhabi launched a *unified health information exchange platform* called Malaffi, which allows approximately 2000 public and private health care providers across the Emirates to access and share information for approximately 3 million people [52]. This top-down approach is very effective when there are adequate funds to enact the process, but not possible in a country similar to the United States, where there is no single authority for a very disparate private health care system.

Belgium has coordinated an interoperable health record for all citizens, which came to full implementation in 2019, called MijnGezondheid [53]. Patient records can now be seen by any physician in any hospital in the country, not an easy feat when considering it includes all periphery hospitals, mental health institutions, pharmacies, and laboratory systems in 2 languages across 3 regions.

## Viewpoint on Best Practices

All stakeholders within a health system can participate in shaping EHRs to be useful and evolved. The following are considerations for establishing best practices for effective and interoperable EHRs.

### Standards

Adopt international standards such as FHIR, LOINC, and SNOMED CT and introduce these standards starting in medical school and university informatics classes. There should be International Standard Organizations standards required of any wearable that is integrated into an EHR so that physicians can be assured the data are reliable. For instance, a 6-m walking test may be performed by a patient at home and recorded for reference, but the results must be obtained by a device that has been proven to have accurate readings in a clinical setting. This is integral to the policy work on digital health regulation.

### Education and Awareness

It is the responsibility of health care administrations to understand interoperability obstacles, the benefits of achieving this, and how it may be done. Investigation is required. In addition, a top-down approach is not the only effective means for adoption of interoperable EHRs. Citizen scientists are constantly developing their own hacks for integration of digital health data, and indeed everyone, from patients to surgeons and from physiotherapists, nurses, to the billing office, should be involved or at least aware of the design process as it affects them all. Use the principle of user experience design and the way that all digital health platforms should be developed: know your user. A software developer may not intuit a cardiologist’s needs (for instance, fast access to images and laboratory results) as opposed to a general practitioner (perhaps most important is an immediate view of history and medications); therefore, physicians, nurses, and administrators must be there to advise and do testing. Physicians can bring solutions that work to hospital administration, highlighting the benefits. On the other side, information technology professionals should be aware of how reimbursement works (among a myriad of other processes) and who needs to see what information when, including the entire arc of care from home caretakers to statisticians.

Ensure awareness of wearables and other sensor data and the fact that eventually patients will likely want this information to be incorporated into their EHR. The new companies developing EHR integration software must also be discerning of the quality and clinical validity of data being integrated.

### Privacy

Hospital administration should request education on privacy and cybersecurity issues, and perhaps, ministries of health should offer short courses to strengthen their knowledge base. Ideally, hospital administration will feel comfortable in considering innovative solutions such as blockchain or in hiring the appropriate people who can, to ensure security and integrity of all patients’ health data.

Hospital administration, ministries of health, and the general public should know how to access their data, how data are protected, and what the data can do for them. Perhaps there can be public service announcements on television, radio, and social media.

### Alternative Solutions

Although the importance of interoperability seems to be a concept now recognized by the large EHR vendors, alternative and economically feasible solutions should be considered by health care administrations. There are solutions that do not require an entire retrofit of a hospital system to deliver data, which avoids the issue of interoperability altogether: Redox, which states it is Health Insurance Portability and Accountability Act compliant and secure, can intake HL7, FHIR, CDA, or X12 data, combine the data, and deliver an output [54].

Seqster is a company that officially entered the marketplace in 2018 and claims to be, “the only technology capable of enabling the majority of 350 million Americans to instantly connect to their EHR(s) along with major fitness/wearable devices, and consumer genetic labs” [55]. They have managed to aggregate and unify health information coming from Epic, Cerner, Strava, and even Fitbit.

There are likely many more companies that will appear in the marketplace as the value is increasingly recognized for having interoperable, clean, and accurate health records that can be data mined for life-saving decision making, research, and public health policy.

### Author's Note

Since the writing of this article, Smart on FHIR has been implemented in over 100 Epic sites, and the trend is continuing.

## Abbreviations

AI: artificial intelligence
EHR: electronic health record
FHIR: Fast Health Interoperability Resources
ICD: International Classification of Diseases
LOINC: Logical Observation Identifiers Names and Codes
MIMIC: Medical Information Mart for Intensive Care
ML: machine learning
